# An Augmented Method for Collecting PLGA Nanoparticles and the Fabrication of 1, 3, 4, 6-Tetra-*O*-acetyl-2-azido-2-deoxy-D-glucopyranose (Ac_4_2AzGlc)-Loaded PLGA Nanoparticles for Efficient and Prospective *in Vivo* Metabolic Processing

**DOI:** 10.3389/fbioe.2022.833456

**Published:** 2022-06-27

**Authors:** Shubham Parashar, Charu Chauhan, Abhiraj Rajasekharan, Jyoti Rautela, Tanya Jain, Kaisar Raza

**Affiliations:** ^1^ Department of Pharmacy, School of Chemical Sciences and Pharmacy, Central University of Rajasthan (CURAJ), Bandar Sindri, Ajmer, India; ^2^ Laboratory of Chemical Glycobiology, National Institute of Immunology (NII), New Delhi, India

**Keywords:** polymeric PLGA nanoparticles, metabolic glycan engineering, RAW 264.7 cell, C57BL/6J, Ac_4_2AzGlc, nanoprecipitation

## Abstract

We investigated two ways for fabricating 1, 3, 4, 6-tetra-*O*-acetyl-2-azido-2-deoxy-D-glucopyranose (Ac_4_2AzGlc)-loaded poly (lactic-co-glycolic acid) PLGA nanoparticles in this article : 1) single emulsion solvent evaporation and 2) the nanoprecipitation method. Among the available methods of collecting nanoparticles using an ultra-high-speed centrifuge, we improvised a less-known method for collecting synthesized nanoparticles without a high-speed centrifuge, based on molecular weight (MW)-dependent centrifugal filters. These nanoparticles were collected in a tabletop centrifuge at a meager centrifugal force in the range of 200–300 xg whereas the conventional high-speed centrifuge method for nanoparticle recovery results in a hard nanoparticle pellet with poor resuspendability which hampers the yield and outcomes of the product. The Ac_4_2AzGlc-loaded PLGA nanoparticles were spherical in shape with consistent and reliable nanometric particle size. The polydispersity indices were well within the acceptable limits. The preliminary studies in RAW 264.7 cell and C57BL/6 mice advocated efficient engineering in the former; however, the latter needs further confirmatory investigations. Preliminary *in vivo* studies with un-encapsulated Ac_4_2AzGlc showed poor engineering of cardiac glycoproteins, opening up avenues for Ac_4_2AzGlc-loaded nanoparticles for improved bioavailability and efficient metabolic engineering.

## 1 Introduction

Applications of nanotechnology in medicine are enormous and can provide essential tools for diagnosing and treating several physiological and pathological conditions. In this context, the development and engineering of polymeric nanoparticles, solid lipid nanoparticles, quantum dots, and other nanomaterials have all been carried out. They can be used in various therapeutic methods, including drug delivery systems, theragnostic, contrast agents, and nanoparticle-based MRI imaging. Biodegradable and biocompatible polymers produced from synthetic and natural materials have been used to create nanoparticles (NP) for drug delivery applications, with these polymers exhibiting effectiveness. Natural polymeric materials are difficult to produce in high purity, but synthetic polymers may be generated in high purity via precise and regulated manufacturing methodologies. Synthetic polymers have been intensively investigated as colloidal materials for the production of nanoparticles as carriers of therapeutic agents. The purity and reproducibility of synthetic polymers are superior compared to those of natural polymers. It is thought that the polyester family would be helpful in the medical field because of their biocompatible and biodegradable characteristics. The use of poly (lactide-co-glycolide) (PLGA) for human therapeutics use has been approved by the Food and Drug Administration (FDA) (De Jong and Borm, 2008; Lewinski et al., 2008; Almeida et al., 2011; Baetke et al., 2015; Wolfram et al., 2015; Boraschi et al., 2017; Wu et al., 2018; Alimardani et al., 2021).

There are two approaches to nanoparticle synthesis, one being the top-down approach wherein we break the bulk material to the one we require, and the other being the bottom-up approach where we assemble our material from building blocks. Nanoparticle preparation can be classified in terms of the phase of medium employed and in terms of the method of monomer preparation. The gas phase, liquid phase, aerosol phase, and solid phase have all been used as a medium for nanoparticle preparation. Some of the widely used methods for nanoparticle preparations are the single emulsion method (oil-water), double emulsion method (water-oil-water), thin layer hydration method (making thin lipid film by removal of organic solvent), and nanoprecipitation method (solvent displacement method). After their formation, the traditional method of nanoparticle collection includes either dialysis or high-speed centrifugation to get rid of unbound/excess polymer/drug and other substrates. However, these frequently used methods come with the following drawbacks, dialysis is a time-consuming process, high-speed centrifugation yields a sturdy nanoparticle pellet, which makes it very difficult to resuspend; furthermore, the use of sonication for resuspension comes with the problem of hampering the shape, the size and polydispersity index (PDI) of the prepared nanoparticles and there is also a potential loss of encapsulated compound which could be the result of hampered integrity of nanoparticles. *Kin Man Au et al.,* presented an improvised method for nanoparticle collection using centrifugal filters, which helps overcome the drawback of the traditional nanoparticle collection method. They used nanoprecipitation method to prepare PEG-PLGA nanoparticles with a hydrodynamic diameter of 100 nm for the therapeutic delivery of BEZ235 (Dactolisib which is an PI3K/mTOR inhibitor) to CD20 and HLA-DR expressing lymphoma cells for targeted therapy, and then purified the resulting nanoparticles using molecular weight (MW) dependent centrifugal filters (Au et al., 2020). Following their protocol of collection and the purification of nanoparticles, we improvised this fabrication method of PLGA nanoparticles encapsulating the monosaccharide analog Ac_4_2AzGlc as a model system.

Non-canonical hexosamine analogs could be used as metabolic glycan reporters by selectively labeling glycans both *in vitro* and *in vivo*, and could be expanded for multiple applications. *Zaro et al.* identified Ac_4_2AzGlc as a metabolic glycan reporter for β-*O*-GlcNAc alterations in diverse mammalian cell lines*.* Once inside the cell, cellular esterases cleave the acetyl groups, and 2-azido-2-deoxy-glucose (2AzGlc) acts as a substrate for biosynthetic enzymes to be incorporated into β-*O*-GlcNAcylated proteins. The incorporation of Ac_4_-2Az-Glc was not found in surface glycoproteins (Zaro et al., 2017). Our research presented here looks at the feasibility of using 2AzGlc to engineer glycans in RAW 264.7 cells (mouse macrophage cell line) and cardiac glycoproteins of C57BL/6J mice, as well as to figure out encapsulation of the same into the synthesized nanoparticles to increase the bioavailability and efficacy of *in vivo* labeling of glycans.

## 2 Materials and Methods

### 2.1 Reagents

Poly (lactic-co-glycolic acid) (PLGA; 50:50; Avg. molecular weight (Mw): 46.6 kDa, avg. molecular weight (Mn): 27.6 kDa, polydispersity (Mw/Mn): 1.69, inherent viscosity (dL/g): 0.65) acid terminated were purchased from EVONIK- LACTEL Absorbable (Cat. B6013-2 R&D grade). CuSO_4_.5H_2_O (Cat. No 102790), acetonitrile (Cat. No 61830025001730), methanol (Cat. No 61860725001730), K_2_CO_3_ (Cat. No 60492005001730), pyridine (Cat. No 82230105001730), acetic anhydride (Cat. No 822278), triethylamine (Cat. No 8.08352.0521), dimethylformamide (DMF) (Cat. No 227056), 4-dimethylaminopyridine (DMAP) (Cat. No 522805), ethanol (Cat. No 100983) and hydrochloric acid (Cat. No 30721) were purchased from MERCK/Sigma. EDC.HCl (Cat. No 0104244) was purchased from Spectrochem. D-Glucosamine hydrochloride was purchased from New Zealand Pharmaceuticals. Amicon^®^ Ultra-15 Centrifugal Filter Unit (Cat. No UFC903096; 30 KDa cutoff) regenerated cellulose membrane, 15 mL sample volume centrifugal filters were purchased from Millipore. Perfektum^®^ Matched Numbered Glass Syringe (Cat. No 0482) by Popper and Sons Inc. was purchased from Sigma Aldrich. Syringe Pumps (Harvard Apparatus-11 Plus; Cat. No 70-2208) were purchased from Harvard Apparatus Inc. Temperature-controlled magnetic stirrers (Model No. IKA C-Mag HS 7 Control and IKA C-Mag HS 7 Digital) were purchased from IKA India, and a refrigerated centrifuge (Model No. 5430R) was purchased from Eppendorf. For TEM sample preparation, 1.0% aq. phosphotungstic acid (Cat No. 15662) was procured from Fisher Scientific. All aqueous solutions were prepared in ultrapure Milli-Q water that had been deionized and filtered (Milli-Q Academic, Millipore, France). Phenomenex C18 column (Cat. No 00G-4252-E0) was utilized in a Shimadzu HPLC with PDA and D2 lamp.

### 2.2 Methods

#### 2.2.1 Synthesis of 1,3,4,6-Tetra-*O*-acetyl-2-azido-2-deoxy-D-glucopyranose (Ac_4_2AzGlc) (1)

**FIGURE 1:**
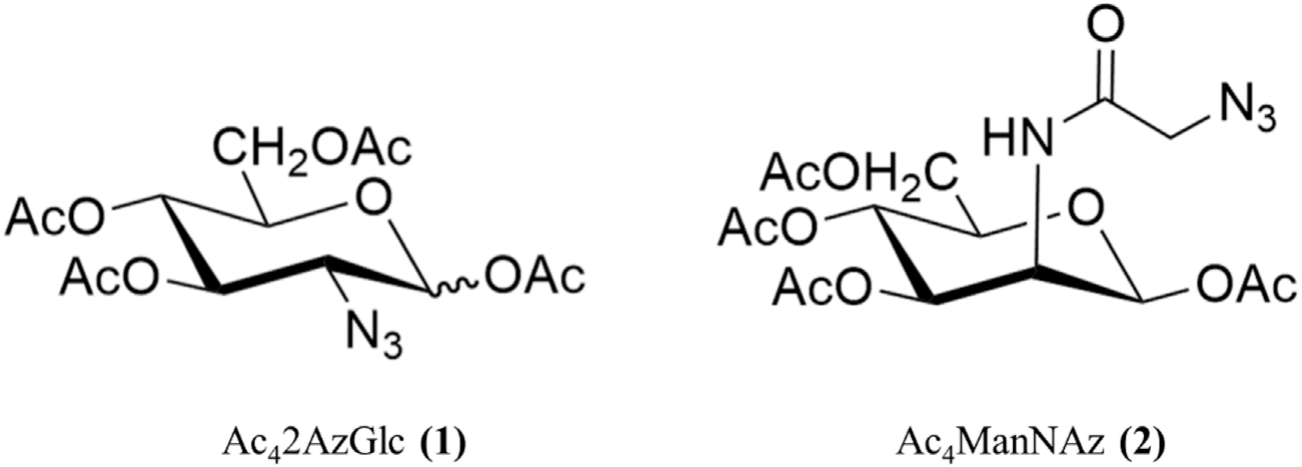
Structure of compound used in the studies. Ac_4_2AzGlc **(1)** and Ac_4_ManNAz **(2).**

1,3,4,6-Tetra-*O*-acetyl-2-azido-2-deoxy-D-glucopyranose (Ac_4_2AzGlc) was synthesized according to the earlier reported procedures (Saxon et al., 2002; Vasella et al., 1991; Corcilius et al., 2013; Zaro et al., 2017)**.** Briefly, in a single quantity, imidazole-1-sulfonyl azide hydrochloride (Goddard-Borger and Stick, 2007) (11.7 g, 55.8 mmol) was added to a suspension of D-Glucosamine hydrochloride (10.11g, 46.89 mmol), K_2_CO_3_ (14.32 g, 103.61 mmol), and CuSO_4_.5H_2_O (130 mg, 0.52 mmol) in 240 mL MeOH at 25°C. The mixture was stirred for 18 h before being evaporated to dryness under a vacuum. Azeotrope with toluene was used to remove any remaining MeOH (2 × 100 mL). The residue was dissolved in a mixture of 240 mL pyridine and 40 mL Ac_2_O with DMAP (cat.), and the suspension was agitated at room temperature for 18 h. The mixture was concentrated *in vacuo*, diluted with 400 mL ethyl acetate (EtOAc), washed with aqueous (3 × 150 mL) 1M HCl, then dried over MgSO_4_ and filtered. The filtrate was concentrated in a vacuum at decreased pressure using a rotary evaporator and purified by silica gel chromatography (Hexane: Ethyl acetate 3:2), yielding an off-white product (8.72 g; 46%). ^1^H, ^13^C, and MS data match with the earlier reported values in the literature. Chemical structure of compound Ac_4_2AzGlc (1) and Ac_4_ManNAz (2) are shown is Figure 1.

#### 2.2.2 Synthesis of 1,3,4,6-Tetra-*O*-acetyl-N-azidoacetylmannosamine (Ac_4_ManNAz) (2)

1,3,4,6-Tetra-*O*-acetyl-*N*-azidoacetylmannosamine (Ac_4_ManNAz) was synthesized according to an earlier reported procedure (Saxon and Bertozzi, 2000; Shajahan et al., 2017). Briefly, 1,3,4,6-tetra-*O*-acetyl-2-amino-2-deoxy-ɑ-D-mannopyranose oxalate was synthesized according to the reported procedure (Sampathkumar et al., 2006). Under argon environment 1,3,4,6-tetra-*O*-acetyl-2-amino-2-deoxy-ɑ-D-mannopyranose oxalate (910mg; 2.08 mmol) and EDC.HCl (940 mg; 4.90 mmol) was dissolved in 20 mL dimethylformamide, following azido acetic acid (680 mg; 6.72 mmol) in 5.0 mL DMF. The reaction was allowed to stir for 1 h, and subsequently, triethylamine (1.5 mL; 10.77 mmol) was added, and the reaction mixture was stirred for 15 h. After the stipulated time, DMF was evaporated under vacuum using toluene as an azeotrope, and the residue was worked up using dichloromethane (DCM) and aq sat. NaHCO_3_ and dried over anhydrous Na_2_CO_3_. The crude mixture was purified over normal phase column chromatography using 230–400 mesh silica gel using (hexane: ethyl acetate 3:2) as mobile phase, yielding white crystalline product (680 mg, 74%). ^1^H, ^13^C, and MS data match with the earlier reported values in the literature. Chemical structure of compound Ac42AzGlc (1) and Ac4ManNAz (2) are shown is Figure 1

#### 2.2.3 Synthesis of 2-Azidoacetic Acid (3)

2-Azidoacetic acid was synthesized according to the reported procedure (Li et al., 2016). Briefly, 13.9 g sodium azide (100 mmol) was dissolved in 60 mL distilled water and chilled to 0°C. Over 10 min, bromoacetic acid (14.30 g, 200 mmol) was added, and the reaction was allowed to gradually warm to room temperature overnight. 5 × 10 mL diethyl ether was used to extract the reaction, acidified to pH = 1. The organic layers were mixed, dried over MgSO_4_, and concentrated to yield colorless liquid 2-azidoacetic acid (5.6 g, 54%). ^1^H data matches with the earlier reported values in the literature.

#### 2.2.4 HPLC Method

Shimadzu HPLC equipped with PDA and D2 Lamps were utilized for reverse-phase high-performance liquid chromatography (RP-HPLC). The samples were run on a Phenomenex C18 column maintained at 40°C and eluants were detected at 210 nm. Unless otherwise noted, the mobile phase contained 100 percent water (A) and 100 percent acetonitrile (B) at an isocratic ratio of (A: B 3:7) with a flow rate of 1.0 mL/min. For all sample injections, a 20 µL loop size was employed.

#### 2.2.5 Preparation of PLGA NPs: Optimization of Methods

For the preparation of PLGA nanoparticles, two methods were employed: 1) single emulsion solvent evaporation and 2) nanoprecipitation method. Various parameters were optimized before selecting the nanoprecipitation method as a method of choice for further experimentation.

#### 2.2.6 Physicochemical Characterization of Nanoparticles

The following metrics were employed to characterize the resultant nanoparticles: size, polydispersity index, zeta potential, and shape. The NPs were recovered by centrifugation (250 xg; 3–4 h unless otherwise stated), and the nanoparticle concentrate was resuspended in ultrapure water, whereas supernatant was collected for evaluation in HPLC to calculate the loading efficiency. The resuspended solution and the filtrate (30 kDa) were both stored for later analysis. The technique of Dynamic Light Scattering (DLS) is well-known for determining the size of nanoparticles in dispersion or in solution. DLS also computes the polydispersity index (PDI), which quantifies the heterogeneity of NP sizes in a suspension. The PDI of a near monodisperse sample should be 0.3 or less. Aggregates can skew the results because the technology treats them as if they were a single huge particle, resulting in a greater PDI. DLS data were measured in this investigation employing a ZetaSizer Nano ZS (Malvern Instruments, Worcestershire, UK). All of the experiments were carried out in disposable 1.0–3.0 mL semi-micro cuvette (Sarstedt) with water as the dispersion medium. Triplicate measurements were taken for each preparation to get meaningful results. Each measurement consists of 12 runs.

#### 2.2.7 *In Vitro* Studies With RAW 264.7 (Murine Macrophage Cell Line)

RAW 264.7 cells were cultured in RPMI medium (Cat No. BE12-702F, Lonza Biosciences) supplemented with 10% heat inactivated-fetal bovine serum (Cat. No. 10082–147, Gibco) and 1% Penicillin-Streptomycin (PS) (antibiotic mixture containing 100 units/mL of Penicillin and 0.1 mg/mL of Streptomycin, Cat. No. 15070, Gibco). For *in vitro* studies, cells were seeded in 100 mm tissue culture-treated plates at a seeding density of 5 × 10^5^ cells/mL in 10 mL medium with either 100 μM of Ac_4_2AzGlc **1)** (free analog), or DMSO (D), or left untreated (U) for 24 h. Post time point, cells were harvested and lysates were prepared in the RIPA buffer (Cat. No. R0278, SIGMA) with PIC (protease inhibitor cocktail, Cat. No. P8340, SIGMA). Lysates were estimated for protein concentration by Bradford assay. To perform click reactions to detect azido functional groups, 200 μg of lysate was clicked with 20 μM DBCO Cy5 (Cat. No. A130, click chemistry tools) for 1 h at 37°C. Proteins were then precipitated by chloroform:methanol: water (1:4:3), and washed with methanol thrice at 16,000 RCF, 5 min. Furthermore, the protein pellet obtained was resuspended in 1% SDS (sodium dodecyl sulfate, Cat. No. RC-930, G Biosciences) and its concentration was estimated. 10 μg of protein was then resolved in a 7.5% SDS-PAGE and Cy5 fluorescence was scanned by Amersham typhoon under a red channel with a pixel size of 100 μm.

## 3 Results and Discussion

### 3.1 NMR Characterization of Ac_4_-2Az-Glc (1) and Ac_4_ManNAz (2)

**FIGURE 2:**
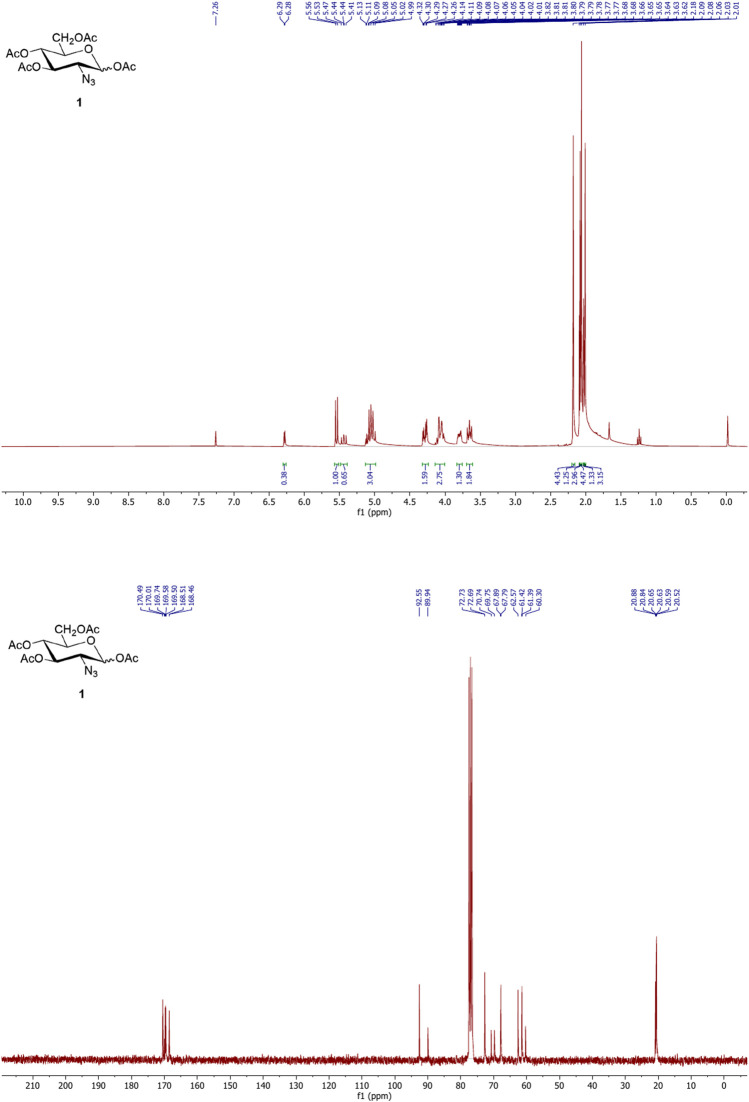
^1^H (upper) and ^13^C (lower) NMR spectra of Ac_4_2AzGlc **(1).**

**FIGURE 3:**
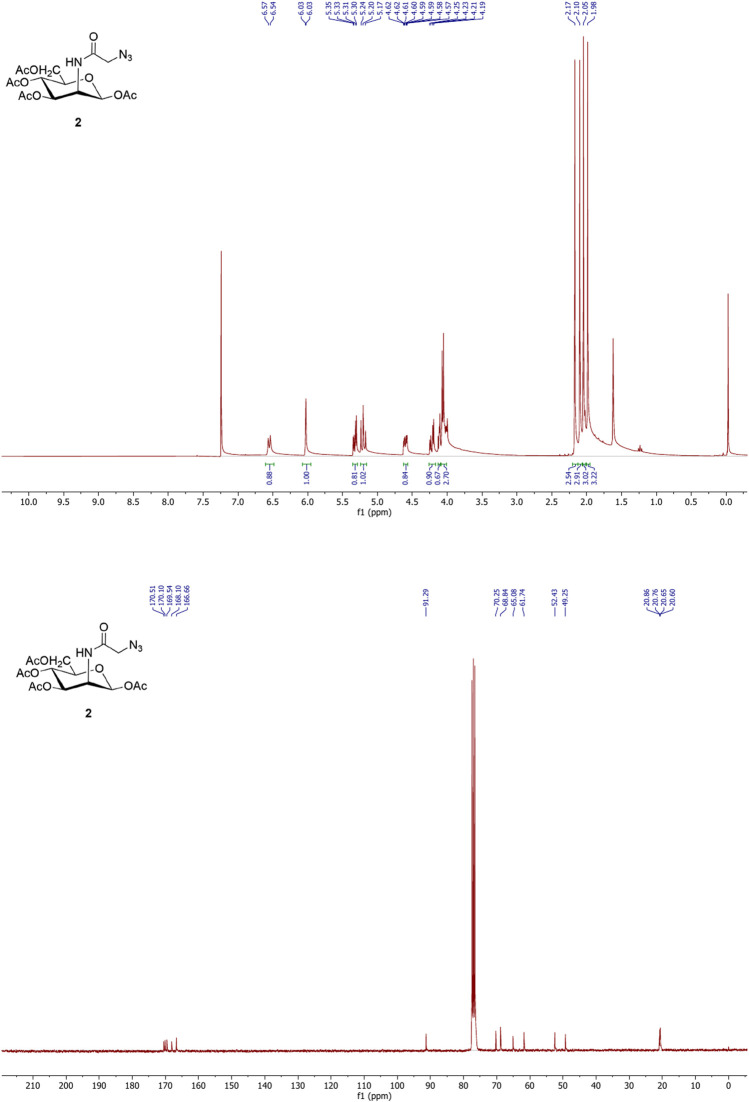
^1^H (upper) and ^13^C (lower) NMR spectra of Ac_4_ManNAz **(2).**

The ^1^H and ^13^C NMR spectra of compounds **(1)** and **(2)** were recorded using a Bruker 300 MHz nuclear magnetic resonance spectrometer. Samples were prepared in chloroform-D (Cat No. 151823, Sigma) with 0.05% v/v TMS as the internal standard. The NMR values of Ac_4_2AzGlc and Ac_4_ManNAz were found to agree with the reported values. The NMR spectra of compounds **(1)** and **(2)** are shown in Figure 2 and Figure 3 respectively.

### 3.2 Synthesis of PLGA-PVA Nanoparticles Using the Single Emulsion Solvent Evaporation Method

#### 3.2.1 Optimisation of Procedure-I

**FIGURE 4:**
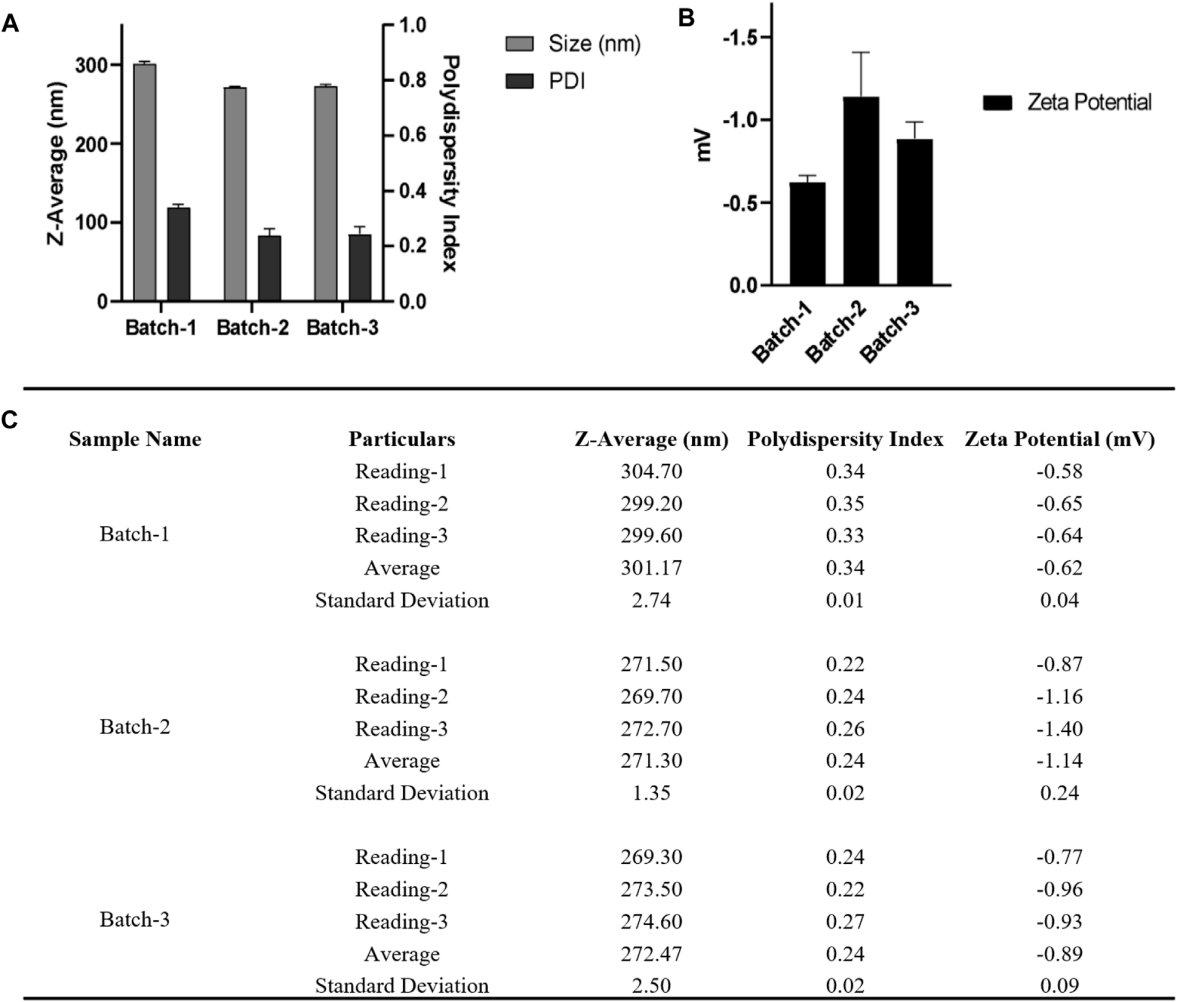
**(A)** DLS and **(B)** zeta-potential measurements for three nanoparticle batches derived using a single emulsion solvent evaporation process with a 50% amplitude and nanoparticle collection at 100,000 xg for 10 min at 4°C **(C)** Table summarizing the different parameters (Size, PDI, zeta potential) of three batches. Bath sonicator was used to resuspend nanoparticle pellet before physicochemical characterization. Error bars represent standard deviations from each runs; *n* = 3.

**FIGURE 5:**
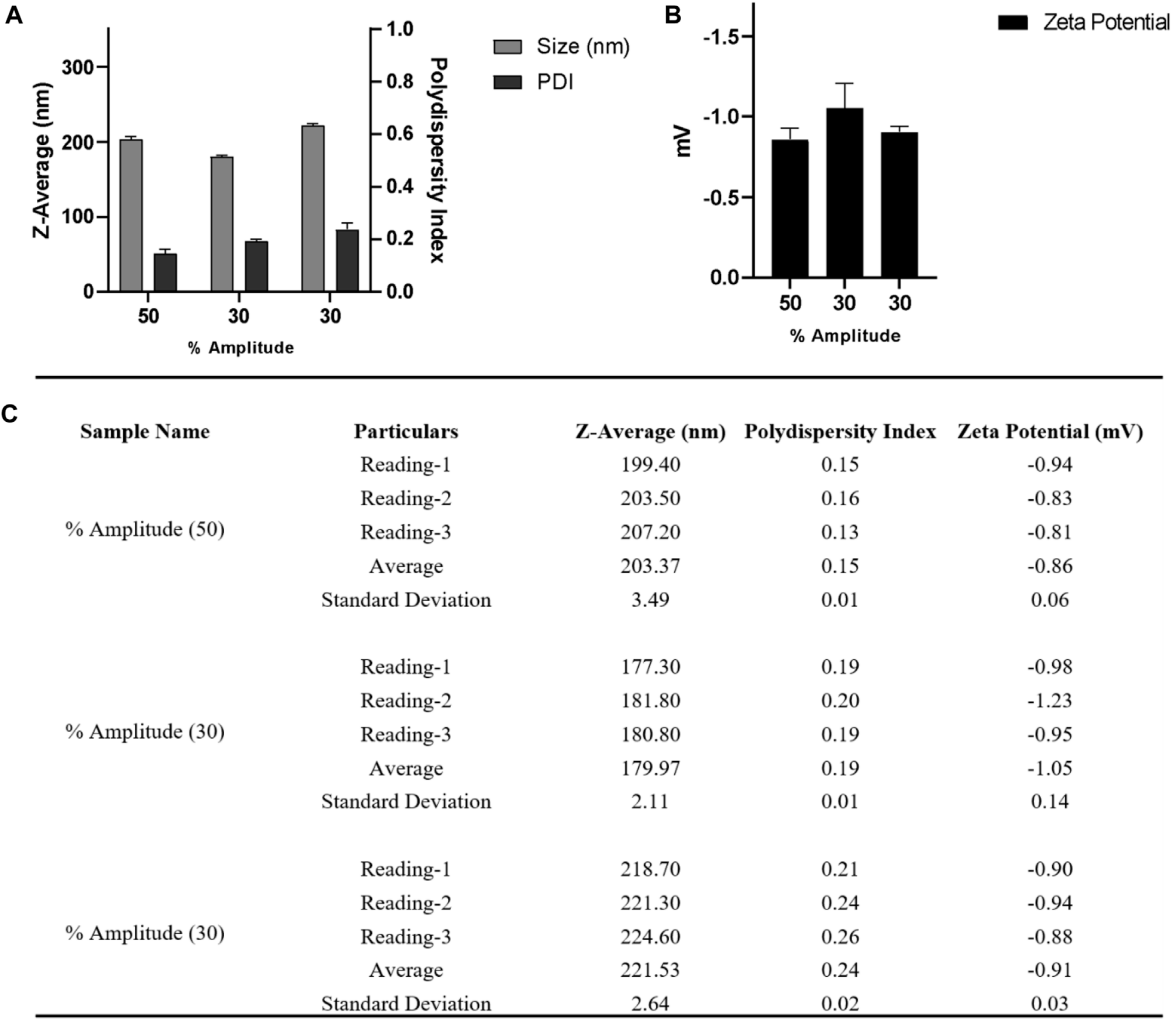
**(A)** DLS and **(B)** zeta-potential measurements for nanoparticle batches obtained from a single emulsion solvent evaporation method after varying the amplitude from 30 to 50% and collecting nanoparticles at 60,000 x g for 20 min at 4°C; **(C)** Table summarizing the different parameters (Size, PDI, zeta potential) of three batches. Bath sonicator was used to resuspend nanoparticle pellet before physicochemical characterization. Error bars represent standard deviations from each run; *n* = 3.

Following a reported procedure (Spence et al., 2015), precisely 20 mg Poly (lactic-co-glycolic acid) (PLGA; 50:50; Avg. molecular weight (Mw): 46.6 kDa, avg. molecular weight (Mn): 27.6 kDa, polydispersity (Mw/Mn): 1.69, inherent viscosity (dL/g): 0.65) acid terminated was dissolved in 500 µL acetone and 300 µL dichloromethane followed by introduction under gentle stirring (1,000 rpm; 25°C) to 3.0 mL ice-water solution containing MgCl_2_.6H_2_O; 45% (w/v) and PVA (30–70 kDa); 2.5% (w/v) in pH 5.0 MES (25 mM) buffer at a flow rate of 0.5–1.0 mL per min. After that, the phases were sonicated employing a probe sonicator for a duration of 90 s at 30 and 50% amplitude, power: 22–7 W (not controlled), Company: Qsonica; Model: q700s on ice. After the sonication was completed, an additional 5.0 mL of 2.5% PVA (30–70 kDa) (w/v) in pH 5.0 MES (25 mM) buffer was introduced under mixing (1,000 rpm; 25°C) to complete the reaction. The samples were allowed to stir for 12 h at 25°C to enable the evaporation of organic solvents. Nanoparticles were spun at 4°C for 10–20 min at varied centrifugal forces ranging from 60,000 xg to 100,000 xg (Company: Beckman Coulter; Model: Optima XPN; Rotor: 70Ti) and repeatedly washed with pH 5.0 MES (25 mM) buffer before being used in the experiments. This optimization of time and centrifugal force were done to evaluate the effect of both parameters in terms of final nanoparticle size and PDI. A bath sonicator is used to resuspend nanoparticle pellets at a final concentration of 5.0 mg PLGA/mL in pH = 5.0 MES (25 mM) buffer, i.e., in 4.0 mL. Figures 4, 5 depict physicochemical characterization of nanoparticle fabrication at 50% amplitude and collection at 100,000 xg and variable amplitude and collection at 50,000 xg.

Following the optimization, the procedure-I was further improved under the following conditions.

#### 3.2.2 Optimization Procedure-II

**FIGURE 6:**
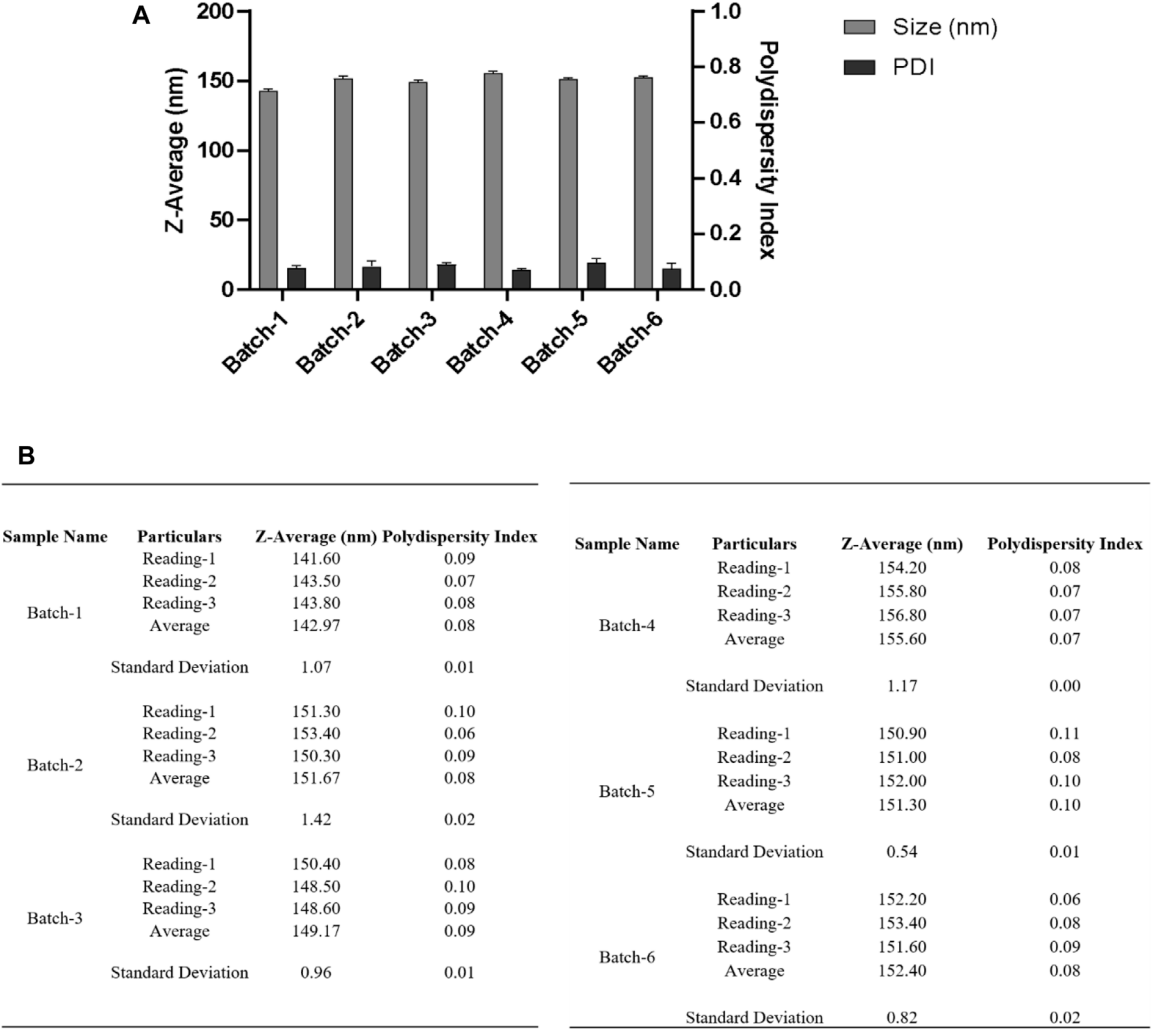
**(A)** DLS measurements for nanoparticles obtained using a single emulsion solvent evaporation method with a 50% amplitude and a collection of nanoparticles at 50,000 xg for 22 min at 4°C. Before measuring DLS, the resuspended nanoparticle pellet was centrifuged at 500 xg to separate any aggregates, and the supernatant was taken for measurement. **(B)** Table summarizing the different parameters (Size, PDI) of six batches. Bath sonicator was used to resuspend nanoparticle pellet before physicochemical characterization. Error bars represent standard deviations from each run; *n* = 3.

Precisely 20 mg poly (lactic-co-glycolic acid) (PLGA; 50:50; Avg. molecular weight (Mw): 46.6 kDa, avg. molecular weight (Mn): 27.6 kDa, polydispersity (Mw/Mn): 1.69, inherent viscosity (dL/g): 0.65) acid terminated was dissolved in 500 µL acetone and 300 µL dichloromethane followed by introduction under gentle stirring (1,000 rpm; 25°C) to 3.0 mL ice-water solution containing MgCl_2_.6H_2_O; 45% (w/v) and PVA (30–70 kDa); 2.5% (w/v) in pH 5.0 MES (25 mM) buffer at a flow rate of 0.5–1.0 mL per min using syringe pump. Following that the mixed phases were sonicated by employing a probe sonicator for a duration of 90 s at "50% amplitude, power: 22–7 W (not controlled), Company: Qsonica; Model: q700s on ice.” After the sonication was completed, an additional 5.0 mL of PVA (30–70 kDa); 2.5% (w/v) in pH 5.0 MES (25 mM) buffer was added under reasonable mixing (1,000 rpm; 25°C) to complete the reaction. The samples were left to stir for 12 h at 25°C to enable the evaporation of organic solvents. Nanoparticles were spun at 4°C for 22 min at centrifugal forces of 50,000 xg (Company: Beckman Coulter; Model: Optima XPN; Rotor: 70Ti) and washed two times repeatedly with pH 5 MES (25 mM) buffer before being used in the experiments. A bath sonicator is used to resuspend nanoparticle pellets at a final concentration of 5.0 mg PLGA/mL in pH = 5.0 MES (25 mM) buffer, i.e., in 4.0 mL. Centrifuged at 500 xg to separate any aggregates, and then the supernatant was taken and analyzed. Samples were investigated without any dilution, as illustrated in Figure 6.

We noticed that the utilization of high centrifugal force for NP collection in a single emulsion solvent evaporation method was the main issue we experienced. The use of high centrifugal force for nanoparticle collection resulted in a compact nanoparticle pellet, which could be due to the presence of polyvinyl alcohol or the use of high centrifugal force for nanoparticle collection. The use of sonication and vortexing to resuspend the resulting pellet may help, but it also jeopardizes the NP physicochemical properties. Furthermore, following sonication, we could not resuspend the pellet completely, so we had to centrifuge it to remove the debris (non-suspended particles). We anticipated that due to these circumstances, we would lose a significant portion of nanoparticle yield and also the use of sonication for nanoparticle resuspension could impair the physicochemical properties of the nanoparticles, leading to the loss of the desired encapsulated molecule.

**FIGURE 7:**
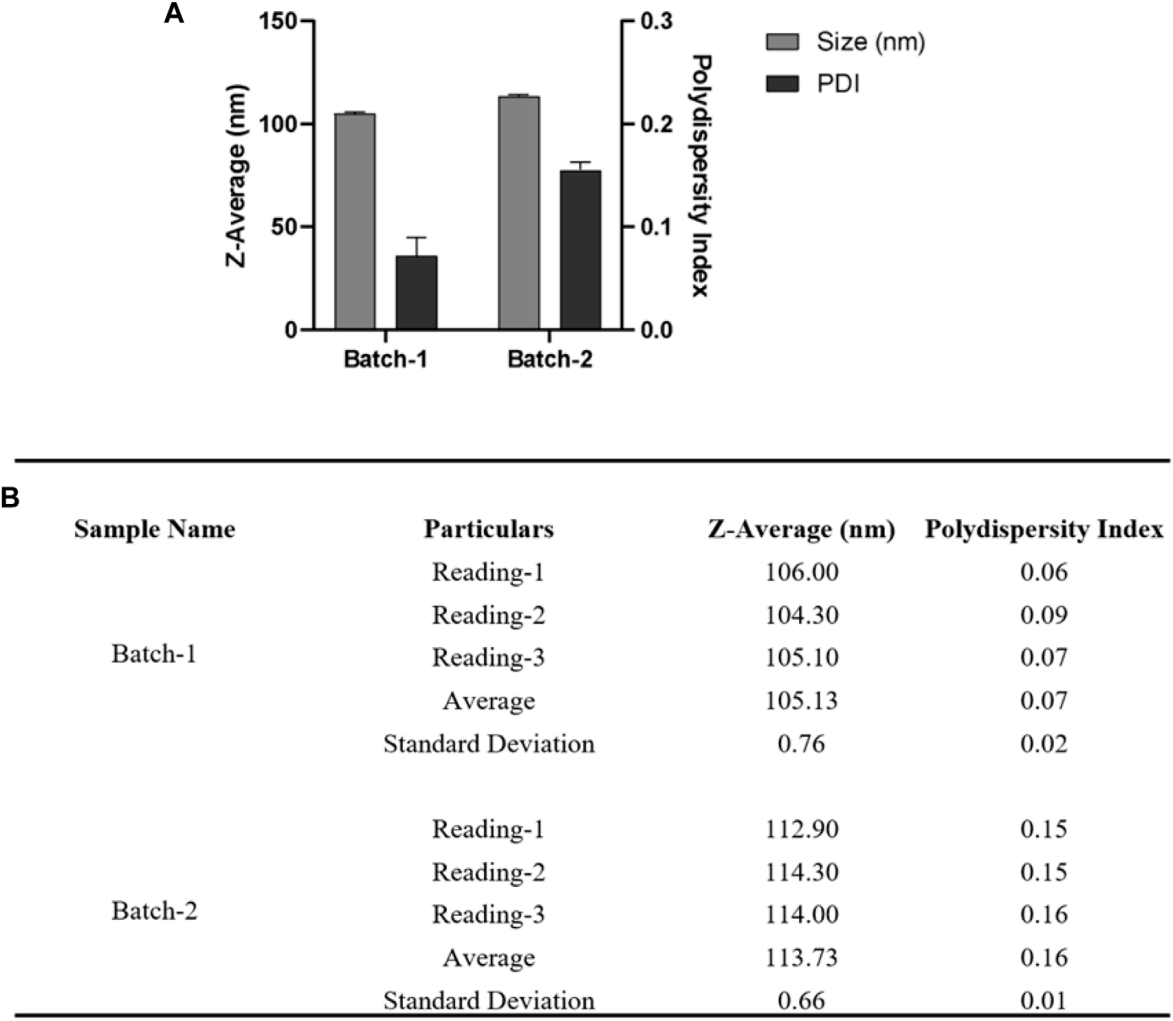
For DLS acquisition, 1.0 mL of resuspended nanoparticles were taken. A total of three individual measurements were taken for each batch, and in each measurement, a total of 13 readings were taken. **(A)** DLS measurements of nanoparticles are shown in a bar graph for two different batches. **(B)** Table summarizing the different parameters (Size, PDI) of six batches. Error bars represent standard deviations from each run; *n* = 3.

To overcome the problem of sturdy pellet and to control other parameters of nanoparticle fabrication, we followed the protocol by *Kin Man Au et al.* (Au et al., 2020). They employed a nanoprecipitation approach to synthesize nanoparticles and used centrifugal filters to remove unbound polymers and collection of the resultant nanoparticles. Herein we followed the nanoprecipitation solvent evaporation technique for the preparation of PLGA nanoparticles. This method is a good choice for obtaining nanoparticles of uniform size with narrow PDI encapsulated with hydrophobic drugs. For the preparation of NP, 30 mg Poly (lactic-co-glycolic acid) (PLGA; 50:50; Avg. molecular weight (Mw): 46.6 kDa, avg. molecular weight (Mn): 27.6 kDa, polydispersity (Mw/Mn): 1.69, inherent viscosity (dL/g): 0.65) acid terminated was dissolved in 3.0 mL acetonitrile and injected at a flow rate of 1.0 mL/min (Harvard Apparatus) under 1000 RPM stirring (IKA) to 12 mL water (milliQ) (ultrapure) and allowed to stir overnight at 25°C. Nanoparticles were then collected using an Amicon filter MWCO 30 KDa (2000 xg for 30 min, unless stated otherwise), washed with water thrice, and then NP pellets were resuspended in 3.0 mL water. Figure 7 depicts the physicochemical characterization of nanoparticles fabricated by the method described above.

**FIGURE 8:**
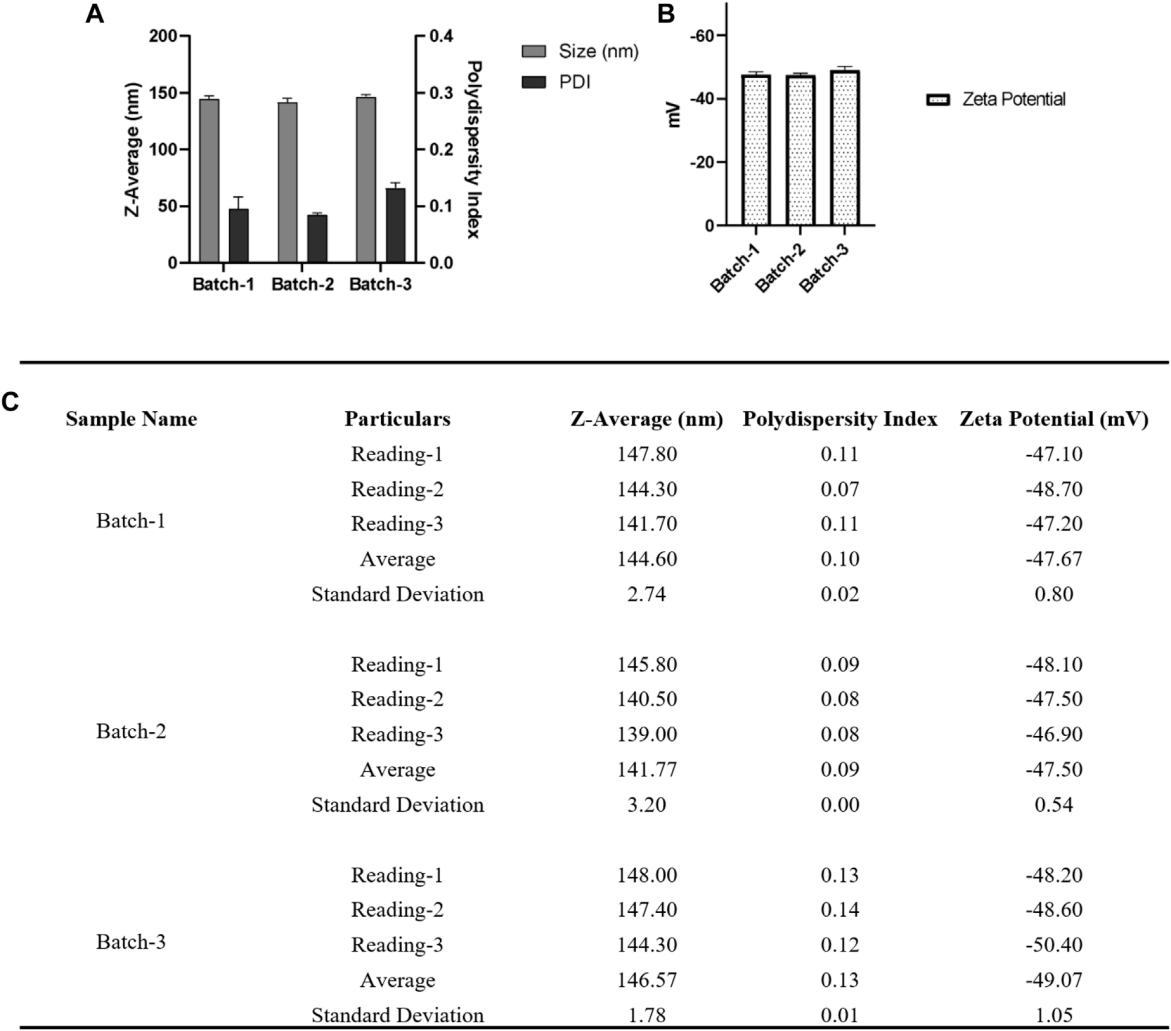
**(A)** For DLS acquisition, 1.0 mL of resuspended nanoparticles were taken and were subjected to a total of three individual measurements for each batch, and each measurement consisted of 12 individual readings. **(B)** For zeta potential, a total of three individual measurements were taken for each batch, and in each measurement, there were a total of 50 individual readings. **(C)** Table summarizing the different parameters (Size, PDI, zeta potential) of three batches. Error bars represent standard deviations from each run; *n* = 3.

We further optimized the procedure to reduce the centrifugal force for the collection of nanoparticles at 2000 xg, and we observed some aggregation of nanoparticle pellet. Hence, we thought to reduce the centrifugal force from 2000 xg to 250 xg, and hence the optimized and improvised nanoparticle fabrication protocol for encapsulation of Ac_4_2AzGlc is as follows. 30 mg PLGA (50:50), Poly (lactic-co-glycolic acid) (PLGA; 50:50; Avg. molecular weight (Mw): 46.6 kDa, avg. molecular weight (Mn): 27.6 kDa, polydispersity (Mw/Mn): 1.69, inherent viscosity (dL/g): 0.65) acid terminated and 10 mg of Ac_4_2AzGlc **(1)** was dissolved in 3.0 mL acetonitrile and injected with a flow rate of 1.0 mL/min (Harvard Apparatus) under 1000 RPM stirring (IKA) to 12 mL water and allowed to stir overnight. Nanoparticles were then collected using an Amicon filter MWCO 30 KDa (250 xg for 30 min), washed with water (1.0 mL) three times and then NP pellet resuspended in 2.0 mL water and subjected to physicochemical characterization as depicted in Figure 8.

**FIGURE 9:**
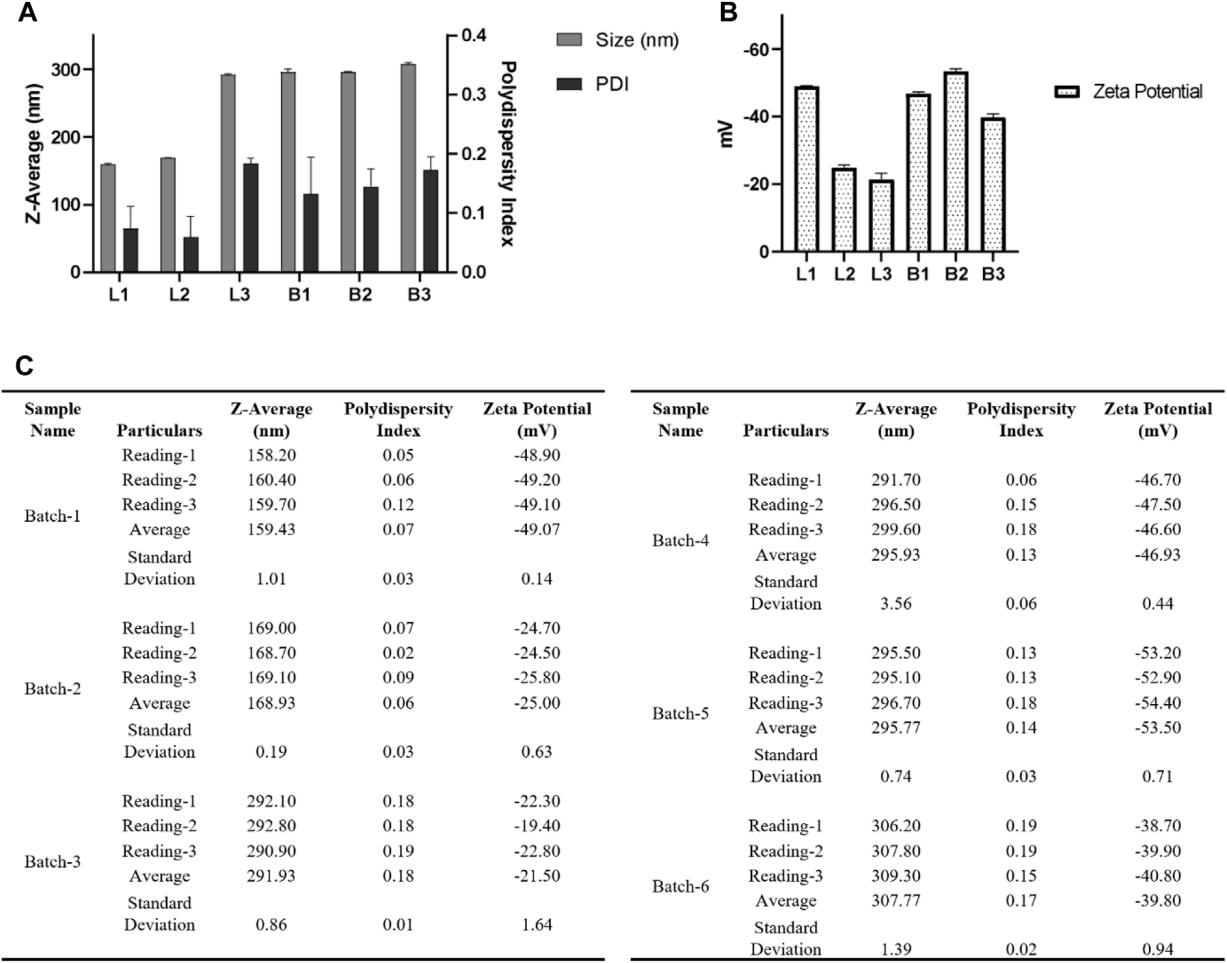
**(A)** For DLS acquisition, 1 mL of resuspended nanoparticles were taken and were subjected to a total of three individual measurements for each batch, and each measurement consisted of 12 individual readings. **(B)** For zeta potential, a total of three individual measurements were taken for each batch, and in each measurement, a total of 50 individual reading was acquired. **(C)** Table summarizing the different parameters (Size, PDI, zeta potential) of six batches. Here L1, L2, and L3 denote three batches of nanoparticles loaded with Ac_4_2AzGlc, and B1, B2, and B3 denote three batches of free nanoparticles without Ac_4_2AzGlc. Error bars represent standard deviations from each run; *n* = 3.

We fabricated three batches of blank and three batches of loaded nanoparticles as per the following: 30 mg PLGA (50:50), Poly (lactic-co-glycolic acid) (PLGA; 50:50; Avg. molecular weight (Mw): 46.6 kDa, avg. molecular weight (Mn): 27.6 kDa, polydispersity (Mw/Mn): 1.69, inherent viscosity (dL/g): 0.65) acid terminated and 10 mg of Ac_4_2AzGlc **(1)** is dissolved in 3.0 mL acetonitrile and injected with a flow rate of 1.0 mL/min (Harvard Apparatus) under 1000 RPM stirring (IKA) to 12 mL of water and allowed to stir overnight. Nanoparticles were then collected using an Amicon filter MWCO 30 kDa (250 xg for 3–5 h or until approximately all the 12 mL was collected, whichever is earlier), and washed three times with 1.0 mL water and then the NPs pellet was resuspended in 2 mL water. Figure 9 represents the DLS and zeta-potential of the fabricated NP by the above method.

### 3.3 HPLC Profile of Ac_4_2AzGlc (1)

**FIGURE 10:**
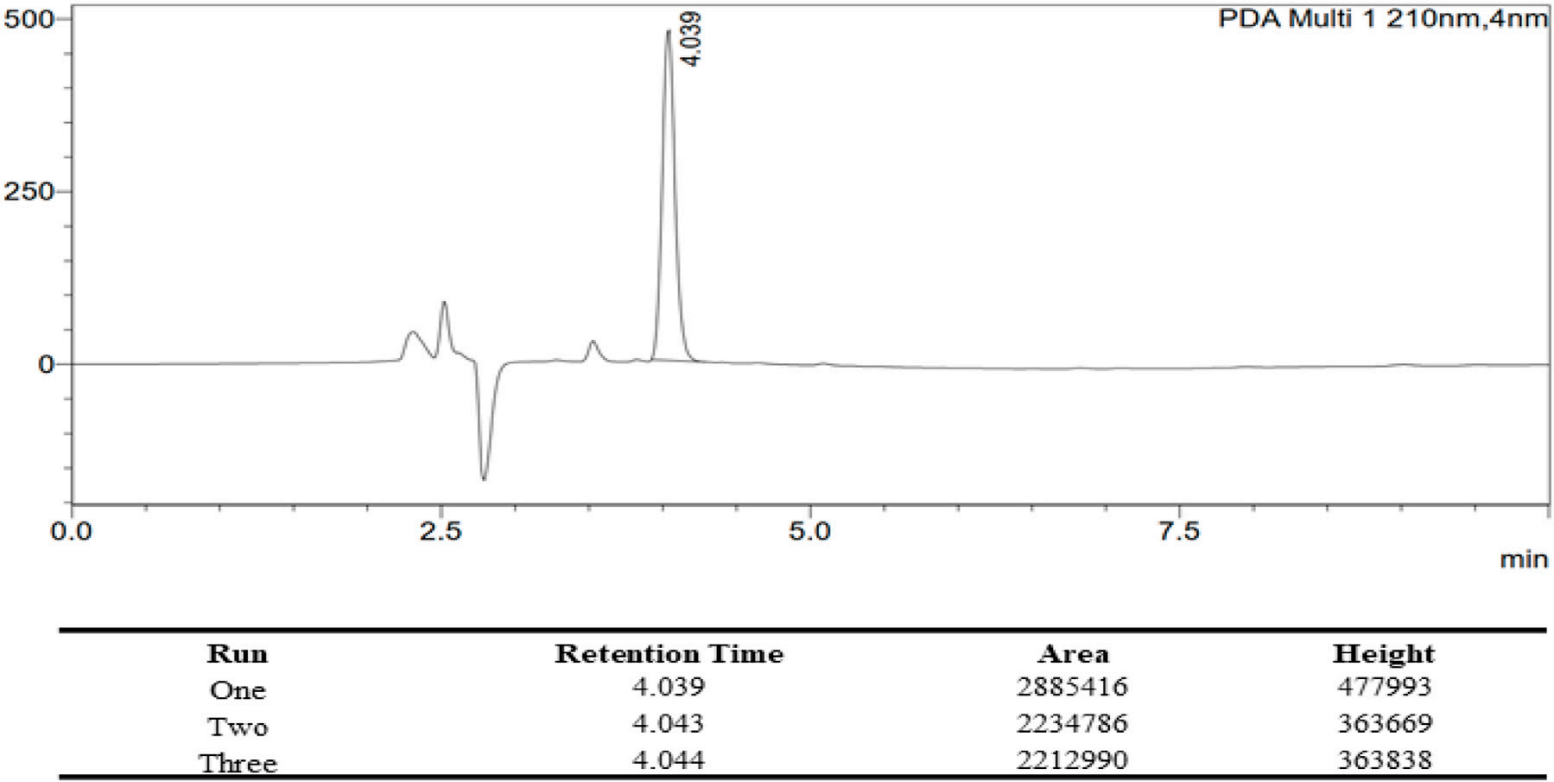
HPLC Profile of Ac_4_2AzGlc (1) with the corresponding table describing the retention time (RT), area under curve (AUC), and height for each individual runs. All the characterization parameters were identical with each run.

Ac_4_2AzGlc stock solution was prepared in ACN: Water (1:1) and then filtered through 0.22 µm PTFE filters before injecting into HPLC equipped with PDA and D2 Lamps for reverse-phase high-performance liquid chromatography (RP-HPLC). The samples were run on a Phenomenex C18 column maintained at 40°C and detected at 210 nm. Unless otherwise stated, the mobile phase contains 100% water (A) and 100% acetonitrile (B) at an isocratic ratio of (A: B 3:7) with a flow rate of 1 mL/min. For all sample injections, a 20 µL loop size was employed. HPLC profile of 20 µL of 1.0 mg/1.2 mL Ac_4_2AzGlc is shown in Figure 10 **.**

### 3.4 Entrapment Efficiency of Ac_4_2AzGlc (1) in PLGA Nanoparticles

In order to calculate the efficiency of encapsulation, we measured the non-encapsulated (leftover) GlcNAc analog in the supernatant. Supernatant collected from individual batches of nanoparticles and Ac_4_2AzGlc (1) were subjected to HPLC, and Area Under Curve (AUC) was determined to calculate the loading of Ac_4_2AzGlc in PLGA nanoparticles as depicted in Table 1 and Table 2 above.

**TABLE 1:** AUC of individual nanoparticle batches and calculation of loading efficiency of Ac_4_2AzGlc in PLGA nanoparticles.

**Sample**	**Height for 20 μL volume injected**	**RT**	**Average of height**
Aca2AzGlc (1.0 mg/1.2 mL)	477993.00	4.03	363669.00
	363669.00	4.04	
	363838.00	4.04	
Loaded nanoparticles TR-1	224020.00	4.05	
	228306.00	4.05	231247.67
	241417.00	4.05	
Loaded nanoparticles TR-2	223498.00	4.05	
	220314.00	4.05	224464.33
	229581.00	4.05	
Loaded nanoparticles TR-3	240320.00	4.04	
	223710.00	4.04	231134.00
	229372.00	4.03	
**Actual amount in supernatant (mg)**	**Volume recovered (mL)**	**Extrapolated height for 12 mL**	**Calculated amount in supernatant (mg) for 12 mL**
10.00	12.00	0.00	10.00
6.36	11.50	0.00	6.64
6.17	11.00	0.00	6.44
6.36	11.00	0.00	6.63

**TABLE 2:** The amount of Ac_4_2AzGlc encapsulated in individual batches of PLGA nanoparticles; encapsulation efficiency was calculated using an indirect method employing HPLC.

Sample	Amount loaded (mg)	Actual amount in nanoparticles (mg)
Loaded nanoparticles TR-1	10	10–6.64 = 3.36
Loaded nanoparticles TR-2	10	10–6.64 = 3.56
Loaded nanoparticlesTR-3	10	10–6.63 = 3.37

### 3.5 Transmission Electron Microscopy of PLGA Nanoparticles Encapsulated With Ac_4_2AzGlc (1)

**FIGURE 11:**
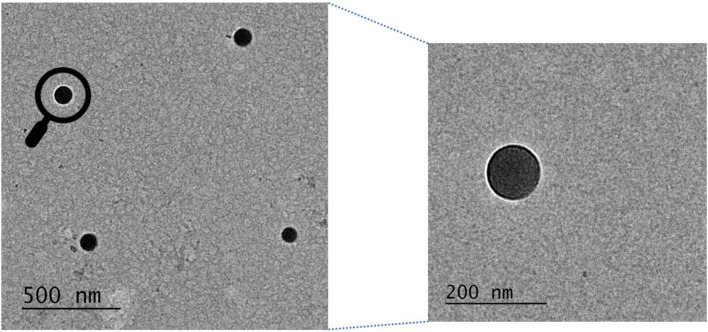
Transmission electron microscopy image of Ac_4_2AzGlc encapsulated PLGA nanoparticles prepared to employ improvised nanoprecipitation method. Zoomed image has also been shown.

Per sample suspension, 10 µL of 1% w/v Alcian blue solution was placed on parafilm. After that, 300 mesh formvar coated copper grids were placed on Alcian blue droplets and incubated for 1 min. The grids were then briefly washed by placing them on four droplets of de-ionized water. After that, 10 µL of nanoparticle suspension was added to the parafilm and then the grid was subjected to the NP suspension and incubated for 10 min. Grids were then washed with de-ionized water and stained with 1.0% w/v aq. phosphotungstic acid for TEM visualization of PLGA nanoparticles (Figure 11).

### 3.6 Metabolic Processing of 1,3,4,6-Tetra-*O*-acetyl-2-azido-2-deoxy-D-glucopyranose Ac_4_2AzGlc (1) in RAW 264.7

**FIGURE 12:**
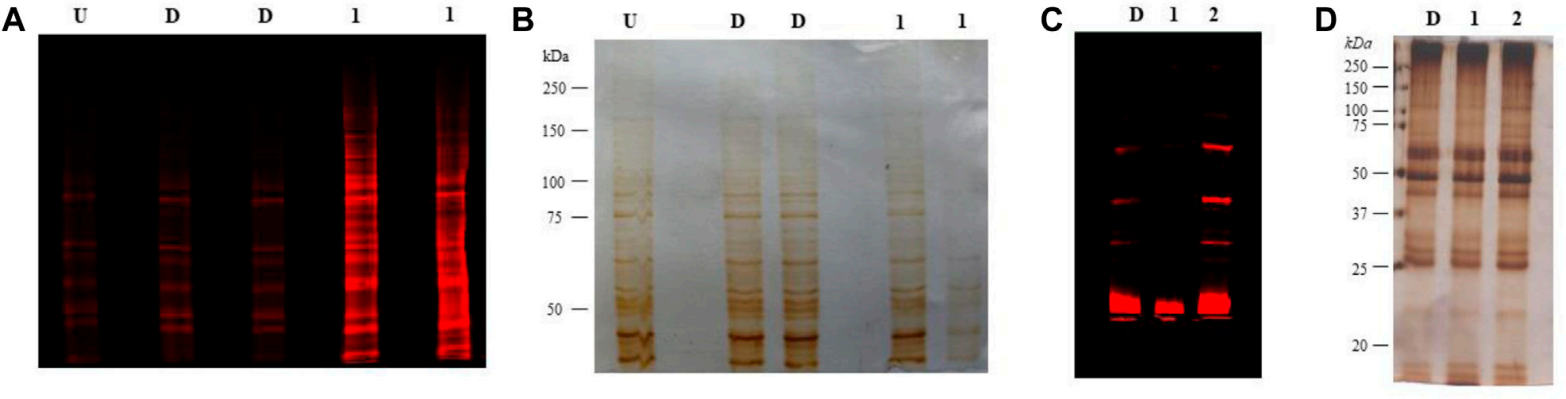
**(A)** RAW 264.7 cells treated with 100 µM Ac_4_2AzGlc (1) and DMSO (control) (D), or left untreated (U) were lysed, clicked with DBCO Cy5, resolved on SDS-PAGE gel, and subjected to fluorescence imaging with corresponding silver staining **(B)** as a loading control. Gels here shown are representative of two technical replicates and biological replicates. **(C)** Heart tissue from C57BL/6 mice *n* = 1 treated with DMSO (control) (D), Ac_4_2AzGlc (1), and Ac_4_ManNAz (positive control) (2) were lysed, resolved on an SDS-PAGE gel, subjected to strain-promoted azide-alkyne cycloaddition (SPAAC) and visualized using fluorescence imaging with corresponding silver staining **(D)** as a loading control. Gels represented here are representative of one technical and biological replicate.

RAW 264.7 total lysates, when treated with Ac42AzGlc (1) for 24 h and probed with DBCO-Cy5, showed engineering of glycoproteins compared to vehicle D, and untreated (U) controls (Figure 12A) and corresponding silver stain gel (Figure 12B). The background signals observed in U and D could be attributed to the non-specificity, or, the notorious nature of the DBCO-Cy5 dye. Metabolic glycan engineering of RAW 264.7 glycoproteins with **(1)** could suggests its incorporation into β-*O*-GlcNAcylated glycoproteins (Zaro et al., 2017). Further enrichment and proteomic studies would be required to confirm and identify the engineered substrates.

### 3.7 Metabolic Processing of 1,3,4,6-Tetra-*O*-acetyl-2-azido-2-deoxy-D-glucopyranose Ac_4_2AzGlc (1) *in Vivo*

Following the successful incorporation of glycoproteins in RAW 264.7 cells, we administered free Ac_4_2AzGlc into C57BL/6 mice (*n* = 1) to investigate if it could engineer the glycans *in vivo*. For 3 days, we injected 300 mg/kg of free Ac_4_2AzGlc (1), Ac_4_ManNAz (positive control) (2), and DMSO (D) (vehicle control) intraperitoneally (analogs dissolved in 90% aq. DMSO for injections), and then on day four mice were euthanized and the heart tissue was harvested after perfusion with ice-cold PBS (5.0 mL) and homogenized using Kinematica homogenizer. The tissue pellet was washed thrice with PBS and then subjected to the TKM lysis buffer (150 mM NaCl, 10 mM Tris, NP-40 1.0% v/v, and protease inhibitor cocktail (PIC), pH 7.4). Lysates were estimated for protein concentration by Bradford assay. To perform click reactions to detect azido functional groups, 200 μg of lysate was clicked with 20 μM DBCO Cy5 (Cat. No. A130, click chemistry tools) for 1 h at 37°C. Proteins were then precipitated by chloroform:methanol: water (1:4:3) (v/v), and washed with methanol thrice at 16,000 rcf, 5.0 min. Furthermore, the protein pellet obtained was resuspended in 1.0% SDS (sodium dodecyl sulfate, Cat. No. RC-930, G Biosciences) and its concentration was estimated. About 4 µg of protein was loaded onto 7.5% SDS PAGE gel, and run for 1.5 h at 100 V, after which the proteins were visualized using an Amersham Typhoon Scanner under the red channel. Our preliminary findings suggest that *in vivo* engineering of glycoproteins in heart lysates was not observed when treated with Ac_4_2AzGlc which could be due to the suboptimal concentration of the compound in systemic circulation or it could be possible that the enzymes in the heart may not process Ac_4_2AzGlc (1) to engineer glycoproteins. The fluorescent signals could be seen in the case of Ac_4_ManNAz (2) injected mice owing to its metabolism in the sialic acid biosynthetic pathway as evident from earlier reports (Saxon and Bertozzi, 2000; Prescher et al., 2004). Background noise/signals can cause minor signals in vehicle control, as seen in strain-promoted click chemistry and sometimes DBCO Cy5 can also give background signals by reacting with cysteine thiols. Figure 12: (c) and (d). To overcome the issue of low bio-availability and targeted delivery of Ac_4_2AzGlc, nano-carriers can be employed, which would be of prospective use to study metabolic glycan engineering *in vivo*.

## 4 Conclusion

In this article, we studied two methods for producing 1, 3, 4, 6-tetra-*O*-acetyl-2-azido-2-deoxy-D-glucopyranose Ac_4_2AzGlc (1) loaded PLGA nanoparticles: 1) single emulsion solvent evaporation and 2) nanoprecipitation. We discovered that the nanoprecipitation method outperformed the single emulsion solvent evaporation method based on size, PDI, and most importantly re-suspensibility of NP pellet. When a high-speed centrifuge was unavailable, we devised a less well-known approach for collecting synthesized nanoparticles. This approach, which depends on MW-based centrifugal filters, was subsequently utilized to collect the nanoparticles in a tabletop centrifuge at a mild centrifugal force in the range of 200–300 xg. When a high-speed centrifuge is used for nanoparticle recovery, a sturdy nanoparticle pellet is created, resulting in aggregated particles with degraded nanoparticle integrity. Approximately 30% encapsulation efficiency was obtained for the nanoparticles encapsulating Ac_4_2AzGlc with the nanoprecipitation method. *In vitro* studies with RAW 264.7 cells showed the effective metabolic engineering of glycoproteins with Ac_4_2AzGlc (1). In our preliminary studies with C57BL/6J mice, we explored the ability of Ac_4_2AzGlc to access and engineer cardiac glycoproteins *in vivo*. Furthermore, we observed that *in vivo* administration of 300 mg/kg Ac_4_2AzGlc (1) did not result in efficient engineering of cardiac glycoproteins by this metabolic precursor, which needs to be further studied by additional investigations. We strongly believe that the use of Ac_4_2AzGlc (1) loaded nanoparticles will further aid in the effective transport and, ultimately, metabolic processing of desired metabolic reporters into glycoconjugates for improved bioavailability and effective metabolic glycan engineering.

## Data Availability

The original contributions presented in the study are included in the article/Supplementary Material; further inquiries can be directed to the corresponding author.
